# Degraded Synergistic Recruitment of sEMG Oscillations for Cerebral Palsy Infants Crawling

**DOI:** 10.3389/fneur.2018.00760

**Published:** 2018-09-18

**Authors:** Zhixian Gao, Lin Chen, Qiliang Xiong, Nong Xiao, Wei Jiang, Yuan Liu, Xiaoying Wu, Wensheng Hou

**Affiliations:** ^1^Key Laboratory of Biorheological Science and Technology of Ministry of Education, Chongqing University, Chongqing, China; ^2^Collaborative Innovation Center for Brain Science, Chongqing University, Chongqing, China; ^3^Department of Rehabilitation Center, Children's Hospital of Chongqing Medical University, Chongqing, China; ^4^Chongqing Medical Electronics Engineering Technology Research Center, Chongqing University, Chongqing, China

**Keywords:** muscle synergy, cerebral palsy, sEMG oscillations, infants crawling, synergistic recruitment

## Abstract

**Background:** Synergistic recruitment of muscular activities is a generally accepted mechanism for motor function control, and motor dysfunction, such as cerebral palsy (CP), destroyed the synergistic electromyography activities of muscle group for limb movement. However, very little is known how motor dysfunction of CP affects the organization of the myoelectric frequency components due to the abnormal motor unit recruiting patterns.

**Objectives:** Exploring whether the myoelectric activity can be represented with synergistic recruitment of surface electromyography (sEMG) frequency components; evaluating the effect of CP motor dysfunction on the synergistic recruitment of sEMG oscillations.

**Methods:** Twelve CP infants and 17 typically developed (TD) infants are recruited for self-paced crawling on hands and knees. sEMG signals have been recorded from bilateral biceps brachii (BB) and triceps brachii (TB) muscles. Multi-scale oscillations are extracted via multivariate empirical mode decomposition (MEMD), and non-negative matrix factorization (NMF) method is employed to obtain synergistic pattern of these sEMG oscillations. The coefficient curve of sEMG oscillation synergies are adopted to quantify the time-varying recruitment of BB and TB myoelectric activity during infants crawling.

**Results:** Three patterns of sEMG oscillation synergies with specific frequency ranges are extracted in BB and TB of CP or TD infants. The contribution of low-frequency oscillation synergy of BB in CP group is significantly less than that in TD group (*p* < 0.05) during forward swing phase for slow contraction; however, this low-frequency oscillation synergy keep higher level during the backward swing phase crawling. For the myoelectric activities of TB, there is not enough high-frequency oscillation recruitment of sEMG for the fast contraction in propulsive phase of CP infants crawling.

**Conclusion:** Our results reveal that, the myoelectric activities of a muscle can be manifested as sEMG oscillation synergies, and motor dysfunction of CP degrade the synergistic recruitment of sEMG oscillations due to the impaired CNS regulation and destroyed MU/muscle fiber. Our preliminary work suggests that time-varying coefficient curve of sEMG oscillation synergies is a potential index to evaluate the abnormal recruitment of electromyography activities affected by CP disorders.

## Introduction

Cerebral palsy (CP) is a permanent movement disorder caused by brain injury in early childhood with a high prevalence of 2–3 per 1,000 live births (1). The regulation of muscle coordination and motor unit (MU) recruitment are usually affected due to the secondary musculoskeletal morphology alterations and changes in the electrophysiological characteristics of MU followed by cerebral injury (2). Surface electromyography (sEMG) signals regulated by central nervous system (CNS) have been widely used to evaluate MU recruitment patterns and neuromuscular function, and it is widely accepted that motor dysfunction of cerebral palsy destroys the synergistic recruitment of muscular activities (3). Although the impact of CP on synergistic electromyography activities has been observed among the muscle group for limb movement (4), very little is known about how motor dysfunction of CP affects the organization of the myoelectric frequency components due to the abnormal motor unit recruitment patterns.

Various features of sEMG signals have been proposed for assessing the abnormal neuromuscular functions in CP patients. The sEMG characteristics from individual muscle, including parameters in time- and frequency-domain, can be used to measure the abnormal overall sEMG activity of CP. Researchers found the changes of magnitude, frequency content and timing from sEMG signals of individuals with CP. The time- and frequency-domain parameters were also utilized to analyze symmetry, cadence, or smoothness of muscle activity for CP patients (5–7). On the other hand, sEMG characters extracted from multiple muscles, such as muscle synergy and co-activation index, can provide the information about relative muscle activation of muscle groups and insights into motor control. Tang et al. (3) reported that CP recruited fewer muscle synergies during upper limb movements with simplified neuromuscular control strategy. Gross et al. (8) found that co-activation index of the rectus femoris/semitendinosus couple was more sensitive to speed, which could be explained by altered motor control. Furthermore, increasing studies suggested that the motor dysfunction caused by CP affected not only the overall sEMG activity but also the sEMG frequency components or oscillations (9, 10).

Recent studies have demonstrated that the sEMG spectrum profile or frequency components correlated with physiological status of movement. Von et al. (11) reported that high-frequency components of sEMG from tibialis anterior were activated before heel strike during running, while low-frequency components were dominated after heel strike. Liu et al. (12) declared that the highest frequency components of sEMG were more sensitive to muscle fatigue than the raw sEMG signal. Moreover, coherence between two sEMG oscillations was used to evaluate the neural control of movement under different conditions, such as low-alcohol and fatigue (13, 14). In addition, studies also reported that coherence can be affected by voluntary force (15). More recent studies employed sEMG oscillation components for abnormal motor functions analysis, and found that the characteristics of sEMG oscillation components correspond to various neuromuscular damages in CP (10, 16). On other hand, sEMG frequency spectrum is correlated the types of recruited MUs, Wakeling et al. have demonstrated that slower and faster MUs in muscles indeed generate low and high sEMG frequency components, respectively (17, 18). Moreover, sEMG oscillations with specific frequency ranges have been successfully used to evaluate the recruitment patterns of corresponding types of MUs in both animal and human muscles during movement (19, 20).

It is generally accepted that multiple element synergistic organization is a fundamental strategy for motor control, and the elements can be well-organized by CNS to perform limb movement (21, 22). A variety of methods have been employed to extract multiple oscillations from sEMG (12, 23, 24), and multivariate empirical mode decomposition (MEMD) shows better performance in decomposing multi-channel sEMG signals due to its self-adaptability, fully data-driven and generalized multivariate extension (16, 25). These studies have indicated that the resulted multi-scale oscillations are mode-aligned across channels, and analysis of multiple oscillations is at the same scale. To obtain the synergistic pattern of multiple elements, although component-based algorithms, such as principal component analysis (PCA) (26, 27) and independent component analysis (ICA) (28), have been introduced to extract synergies, non-negative matrix factorization (NMF) is a better choice (29, 30) due to its non-negative constraint for all the matrices (the raw matrix and the obtained matrices). A few works have demonstrated that NMF can be used to extract muscle synergies from multi-channel sEMG signals in upper limb muscles during motion tasks (31), even to obtain activation patterns of muscle-tendon units and time-vary coefficient curves from high-density sEMG signals during dynamic motion tasks (32).

Inspired by muscle synergy, different types of MUs might also be manifested as specific oscillation synergy patterns. In other words, the synergistic recruitment mechanism should be manifested with coordinated activation of muscle groups and of different types of MUs in individual muscles. Hence, we assumed sEMG frequency components or oscillations might also be recruited with a synergistic pattern for normal motor function, whereas motor dysfunction of CP may affect the organization patterns of oscillation components. To this end, we recorded the sEMG signals from biceps brachii (BB) and triceps brachii (TB) muscles of the upper limbs during infant crawling, extracted sEMG oscillations and analyzed their synergy patterns to explore the abnormal organization of sEMG oscillations in CP.

## Method

### Participants

Under the approval of children's hospital of Chongqing medical university, 17 infants with typical development (TD, 11.4 ± 1.7 months, 12 males and 5 females) and 12 infants with cerebral palsy (CP, 22.3 ± 5.5 months, 9 males and 3 females) were recruited in this study. Cerebral palsy infants were collected from the department of rehabilitation center in the children's hospital of Chongqing medical university. The TD infants were recruited from local child health clinics. The inclusion criteria for CP infants included: (1) all CP infants were under the age of 3 years old; (2) crawling continuously on their hands and knees during the test; (3) no other diseases that lead to motor function deficits according to the historical records. The TD infants were born at term with normal birth weight and had no neurological impairment. To make sure all subjects can crawl continuously on hands and knees, the Gross Motor Function Measure (GMFM-88) was conducted before experiments. Informed consent forms were obtained from participants' legal guardians before experiments.

### Experimental protocol

The infants were encouraged to crawl at their own pace from one end of a mat (360 × 120 cm) to the opposite side. Valid crawling cycle sequence requires infants to crawl continuously on hands and knees more than four cycles. Before the experiment, infants crawled on the mat several minutes to warm up.

**Figure 1:**
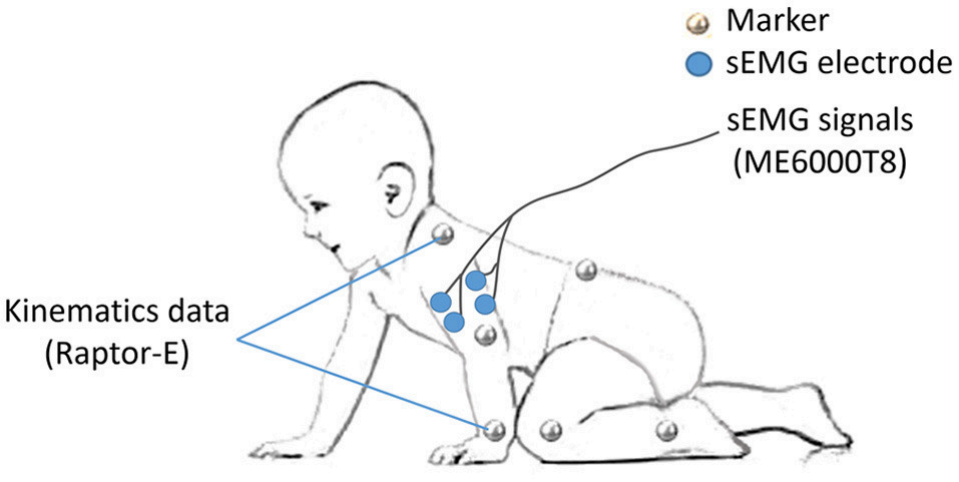
Experiment setup of markers and sEMG electrodes.

As the major activated muscles of the upper limb during crawling, bilateral BB and TB were selected for sEMG recording. After disposable surface electrodes were attached to the muscle belly and bandaged to reduce motion artifacts (Figure 1), sEMG signals were collected using a sEMG system (ME6000T8, Mega Electronics Ltd, Finland) with a 15–500 Hz bandwidth and a 1,000 Hz sampling rate.

To specify the crawling phase, 14 markers were attached on the bony landmarks of the bilateral wrist, elbow, shoulder, hip, knee, ankle, the right spine scapula, and the trunk, respectively (Figure 1). The kinematic data of infants were recorded at 100 frames/s by a 3D motion capture system (Raptor-E, Motion analysis corporation, USA) with six high-speed digital cameras. Kinematic data and sEMG data were stored in a desktop computer and a laptop computer respectively synchronized offline.

### Data analysis

**Figure 2:**
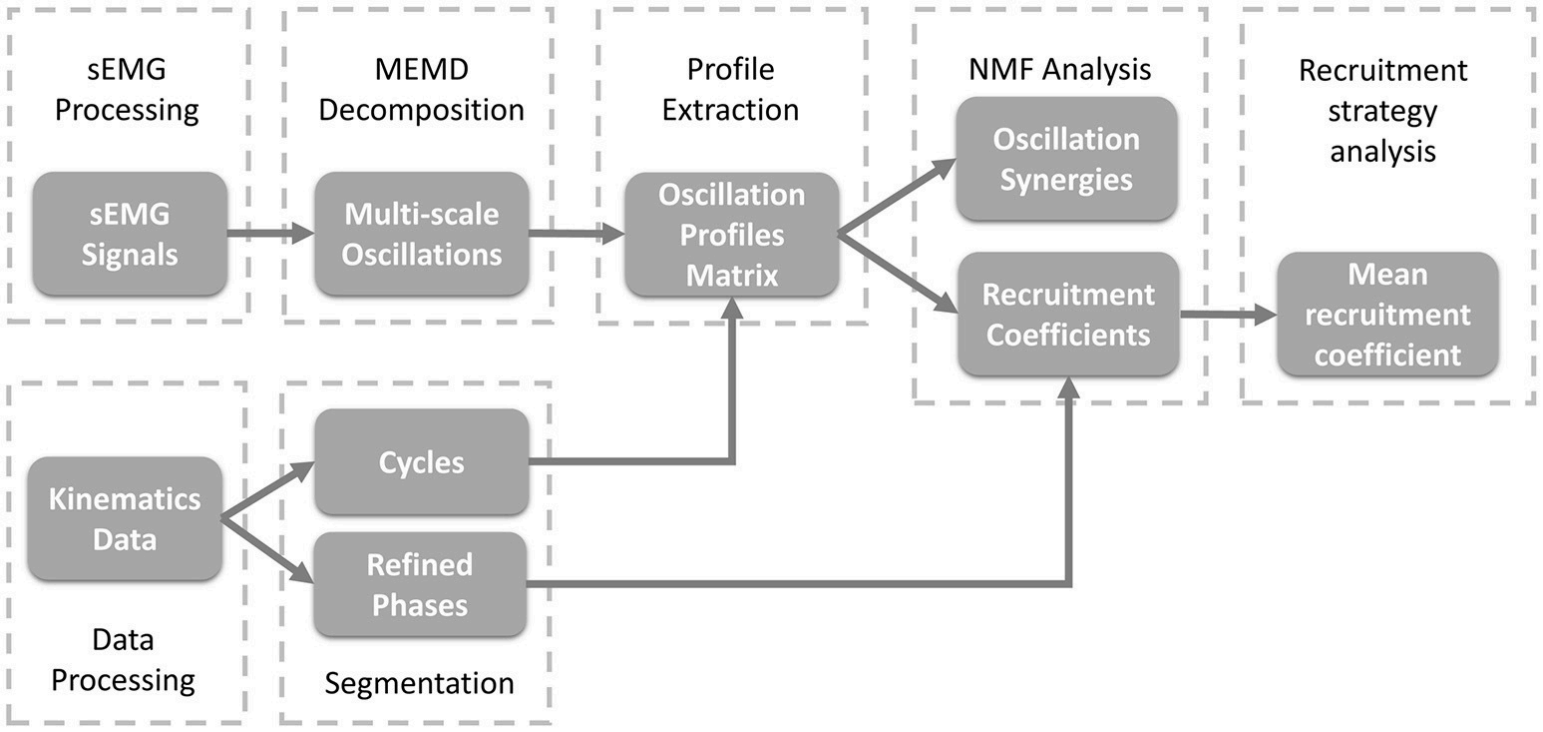
Block diagram of the proposed oscillation synergy analysis framework.

As shown in Figure 2, to study the organization of sEMG oscillations during crawling, multivariate empirical mode decomposition (MEMD) was employed to extract the multi-scale myoelectric oscillations, and non-negative matrix factorization (NMF) was used to analyze the patterns of synergistic organization within sEMG oscillations. Then, the recruitment coefficients within each refined crawling phase were evaluated.

#### Pre-processing

**Figure 3:**
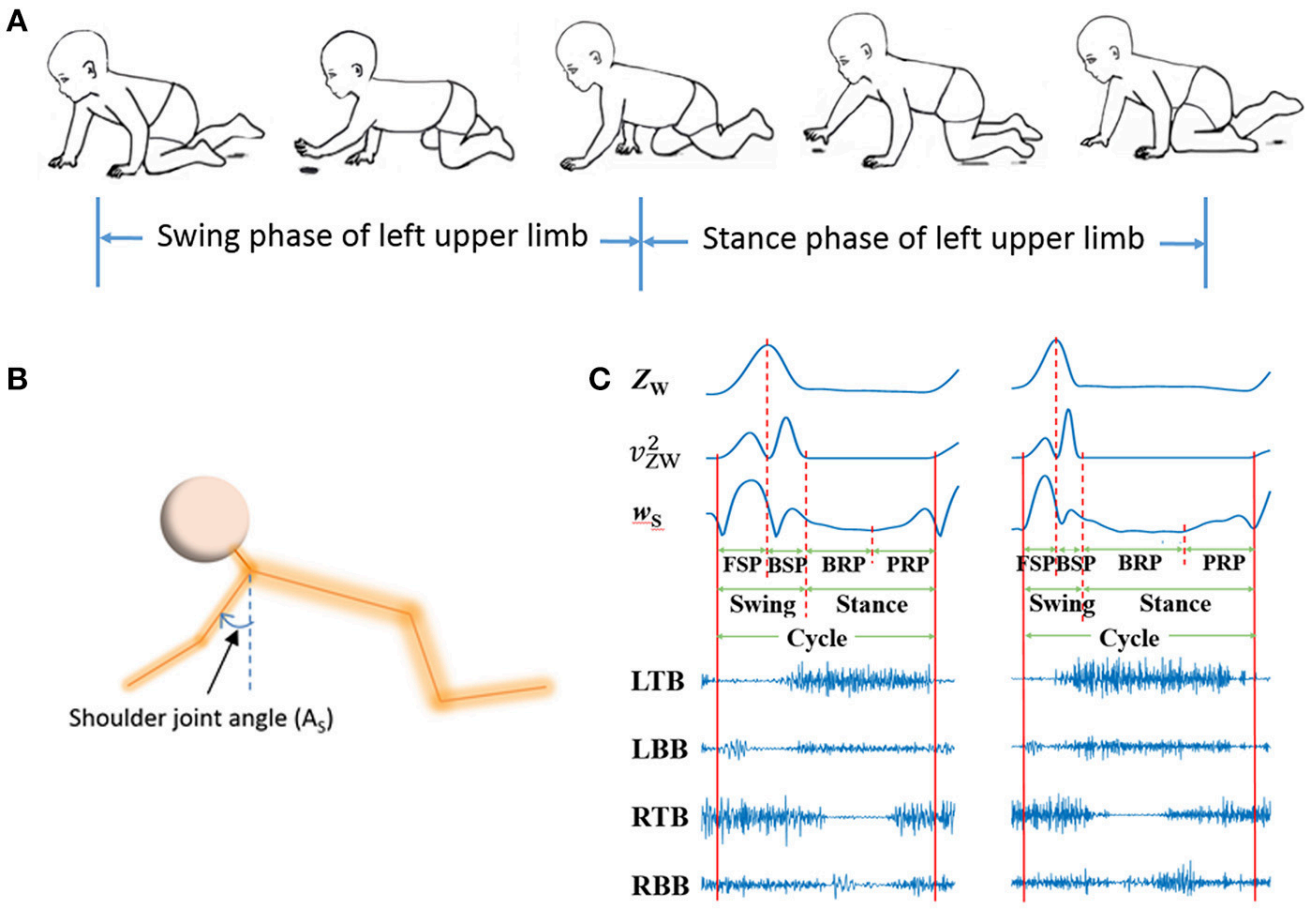
sEMG and kinematic recorded in infant crawling. **(A)** Diagram of the crawling cycle of left upper limb [Adapted from Patrick et al. (33)]; **(B)** Schematic of the shoulder joint angle (AS); **(C)** Refined crawling phases, and the corresponding sEMG signals of bilateral TB and BB (LTB, left TB; LBB, left BB; RTB, right TB; RBB, right BB;) of one crawling cycle for TD (left graph) and CP (right graph).

Firstly, the valid data of more than four consecutive crawling cycles were segmented from raw signals, and sEMG for the BB and TB of both sides and kinematic data were simultaneously recorded (see Figure 3). Then, the valid sEMG signals were processed with a zero-lag high-pass filter (4th order Butterworth filter, 20 Hz). Then, a 50 Hz notch filters were adopted to remove power frequency noise.

#### Oscillation extraction with MEMD

EMD is a fully data-driven analysis approach which self-adaptively decomposes a non-linear and non-stationary signal into several intrinsic mode functions (34). However, for multi-channel signals, applying EMD to each channel could produce a different number of misaligned IMFs. Rehman et al. (35) proposed the MEMD method to extend EMD to multivariate signal decomposition. They treated n-variable time series as n-dimensional vectors and employ the spherical coordinate system to project n-dimensional vectors along different directions in (n-1)-dimensional space, and the mean value of the envelopes of these n-dimensional projection sequences is obtained as the local mean of multiple time series. After MEMD decomposition, the n-dimensional raw signal ${\left\{v\left(t\right)\right\}}_{t=1}^{T}=\left\{v_{1}\left(t\right),v_{2}\left(t\right),\cdots v_{n}\left(t\right)\right\}$ can be decomposed into *d* layers multivariate IMFs *h*_*i*_(*t*) and residual *r*(*t*), which can be described as

(1) $$\begin{array}{r} v\left(t\right)=\overset{d}{\underset{i=\text{1}}{\sum }}h_{i}\left(t\right)+r\left(t\right) \end{array}$$

The IMFs obtained after MEMD analysis have the same number for each channel and orderly align scales across channels (35).

**Figure 4:**
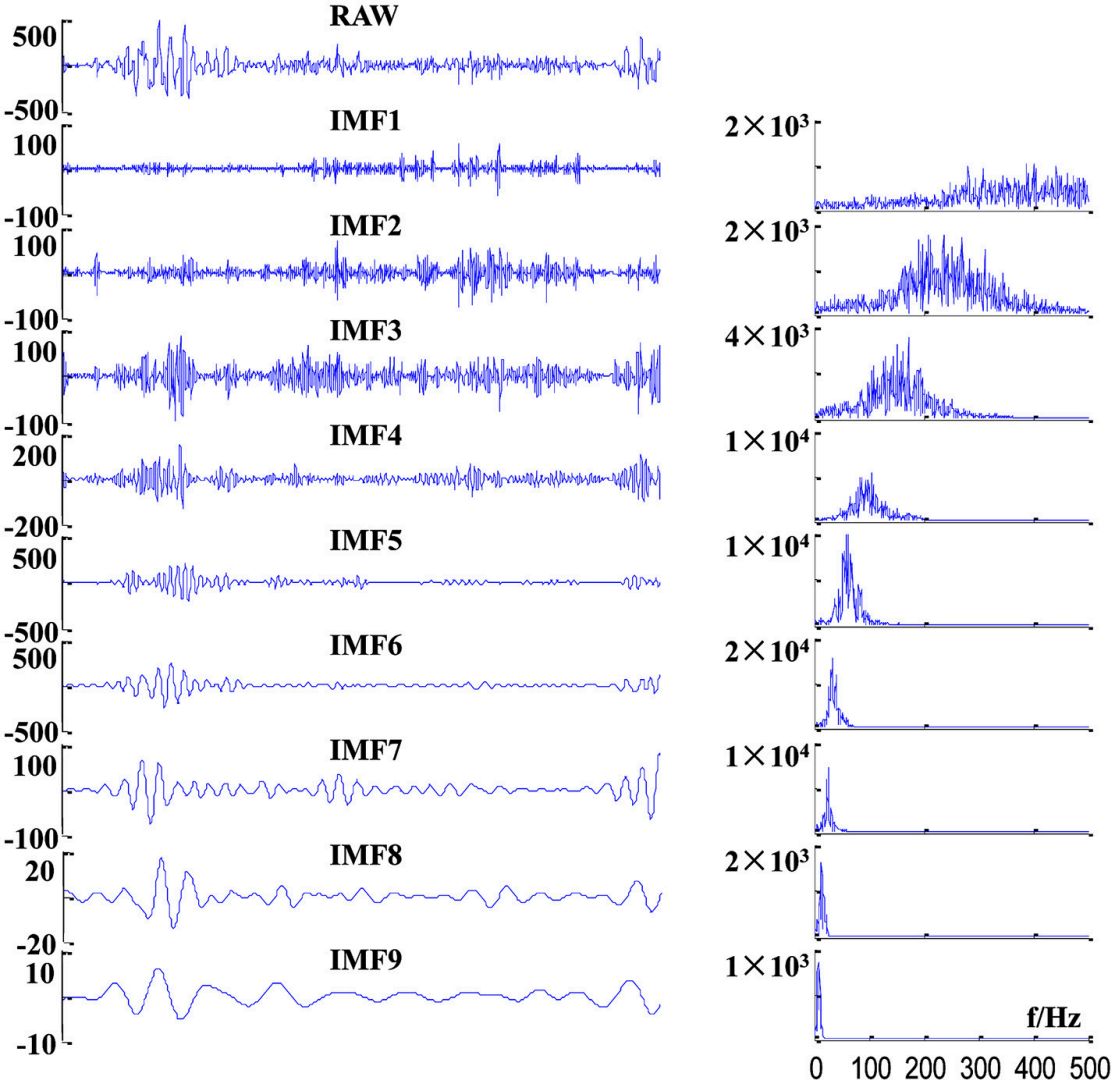
Examples of decomposition results of a raw sEMG signal of BB from one TD subject. (Left panel) sEMG signal and its first nine IMFs, and (right panel) corresponding power spectra of these IMFs.

Referred literature (16), we firstly concatenated the valid sEMG signal segments from all subject as a 4-channel sEMG dataset D, which is shown as following formula:

(2) $$D=\left[\begin{array}{c} CH_{11}-CH_{12}-\cdots -CH_{1j}\\ \vdots \\ CH_{i1}-\;CH_{i2}\;-\cdots -CH_{ij} \end{array}\right]$$

where *CH*_*ij*_ is the *i*th channel of the valid sEMG signal segments for the *j*th subject. Here, we recorded four channel sEMG signals, and totally 29 subjects conducted the test; that is to say, *i* = 1, 2, 3, 4; *j* = 1, 2, …, 29. To reduce the mode mixing in multivariate IMFs (36), 2-channel Gaussian white noise (with the same length as D) were added to the 4-channel sEMG dataset D to composite a 6-channel dataset. Then, the composite dataset was decomposed by MEMD, which yield a total of 23 IMFs with aligned scales across channels, cycles and subjects. Example of raw sEMG signals and their first nine IMFs of BB in a crawling cycle are illustrated in Figure 4, of which the right panel show the corresponding power spectra for raw sEMG and IMFs. The frequency ranges of the first nine IMFs are listed in Table 1, the frequency band ranges of IMFs decrease orderly, and corresponding IMFs for different subjects are scale-aligned.

**Table 1:** Frequency ranges of first nine IMFs determined by 3dB bandwidth.

**IMF**	**1**	**2**	**3**	**4**	**5**	**6**	**7**	**8**	**9**
**Frequency range (Hz)**	284–500	183–352	127–252	81–189	52–116	34–69	23–41	16–24	4–18

#### Kinematics data preprocessing and crawling phase segmentation

Kinematics data were processed with a zero-lag low-pass filter (4th order Butterworth filter, 4Hz) to remove high-frequency noise. As shown in Figures 3A,B, crawling cycle phases was determined with the z coordinates of the wrist (Z_W_) and the shoulder joint angle (the joint angle in sagittal plane) (37). Figure 3C showed the examples of segmentation from two subjects during one crawling cycle. The squared of time derivative of Z_W_ ($v_{Z_{W}}^{2}$, velocity squared) was applied to segment swing and stance, and the crawling cycle begins with swing. A threshold of $v_{Z_{W}}^{2}$ was set at 0.5 (m^2^/s^2^) to decide the onset of limb moving and the end moment of a crawling phase (38). To refine the crawling cycle in detail, the swing phase was divided into forward swing phase (FSP) and backward swing phase (BSP) with the maximum of Z_W_; and stance was divided into braking phase (BRP) and propulsive phase (PRP) with the minimum of time derivative of A_S_ (*w*_S_).

#### Synergistic recruitment analysis of sEMG oscillations

As the frequency of IMF9 is below 20 Hz and out of sEMG frequency range (20–500 Hz), the first eight IMFs are included for oscillation recruitment analysis. Envelopes of the first eight IMFs of each channel sEMG signal were extracted (Hilbert spectrum and median filtering) and segmented into cycles according to kinematics data. The envelopes were re-sampled into 200 points for each cycle. The synergies of sEMG oscillation were extracted with NMF, which decompose a non-negative matrix V into two non-negative matrixes including a base matrix W and corresponding recruitment coefficient matrix C (39). Here, eight envelopes of IMFs resulted from a channel of sEMG within a crawling cycle composed the non-negative matrix V. The NMF method can be described as following

(3) $$V^{m\times n}\;=\;W^{m\times s}C^{s\times n}$$

In this study, the matrix of *V*^*m*×*n*^ represents the envelopes of *m* IMFs (*m* = 8), and *n* is the sample number (*n* = 200). Each column of *W*^*m*×*s*^ represents an oscillation synergy with *m* weight factors (1 ≤ *s* ≤ *m*), and *s* is the number of synergies. Each row of *C*^*s*×*n*^ represents corresponding recruitment coefficients, which shows how each synergy is modulated over time. To maintain the modulation of information within oscillation synergies, C was normalized to its maximum values of the crawling cycle.

The number of oscillation synergies was determined by calculating the variation accounted for (VAF) between the original matrix *V* and the reconstruction matrix *V*′ = *WC* for each *s* value (from 1 to 8) (40). VAF is calculated as follow

(4) $$VAF\;=\;1-\frac{{\left(V-V^{\prime }\right)}^{2}}{V^{2}}$$

Here, the selection criteria were that the mean of VAFs was larger than 95% and the increment of VAF was < 1%.

### Statistical analysis

The recruitment coefficient values for each oscillation synergy were averaged over 3–4 valid cycles for each subject. For each TD infant or CP infant whose both side limbs were affected, as the symmetric movement for left and right side, the oscillation synergy and recruitment coefficient of sEMG signals recorded from right and left BB and TB muscles have been averaged firstly. In order to compare the recruitment pattern of each oscillation synergy, independent sample *t*-test was applied on the recruitment coefficients for each phase. The significance level was set to 0.05. All the data were analyzed in SPSS 22.0 statistical software (SPSS Inc., USA). In addition, Pearson's correlation coefficient (r) between any two oscillation synergies with the same order number was calculated to assess the similarities of oscillation synergy structures. Four kinds of r values were calculated between same within-groups muscles (e.g., BB of TD1 vs. BB of TD2), between different within-groups muscles (e.g., BB of TD1 vs. TB of TD2), between same between-groups muscles (e.g., BB of TD1 vs. BB of CP1) and between different between-groups muscles (e.g., BB of TD1 vs. TB of CP1).

## Results

All of the recruited infants in this study can crawl continuously on hands and knees more than 4 valid cycles (TD, 4.9 ± 0.9 cycles; CP, 5.9 ± 1.4 cycles). Their hands-and-knees crawling scores were at least 44 (TD, 50.9 ± 3.3; CP, 63.7 ± 8.5) according to GMFM. Although the crawling motor function scores of CP infants equal or even exceeded TD infants (in this study) after rehabilitation training, their motor development was indeed slower than that of TD infants at the same age. CP infants exhibited abnormal movement behavior and sEMG activities.

### Three stable oscillation synergies with low-, medium- and high-frequency ranges in muscles

**Figure 5:**
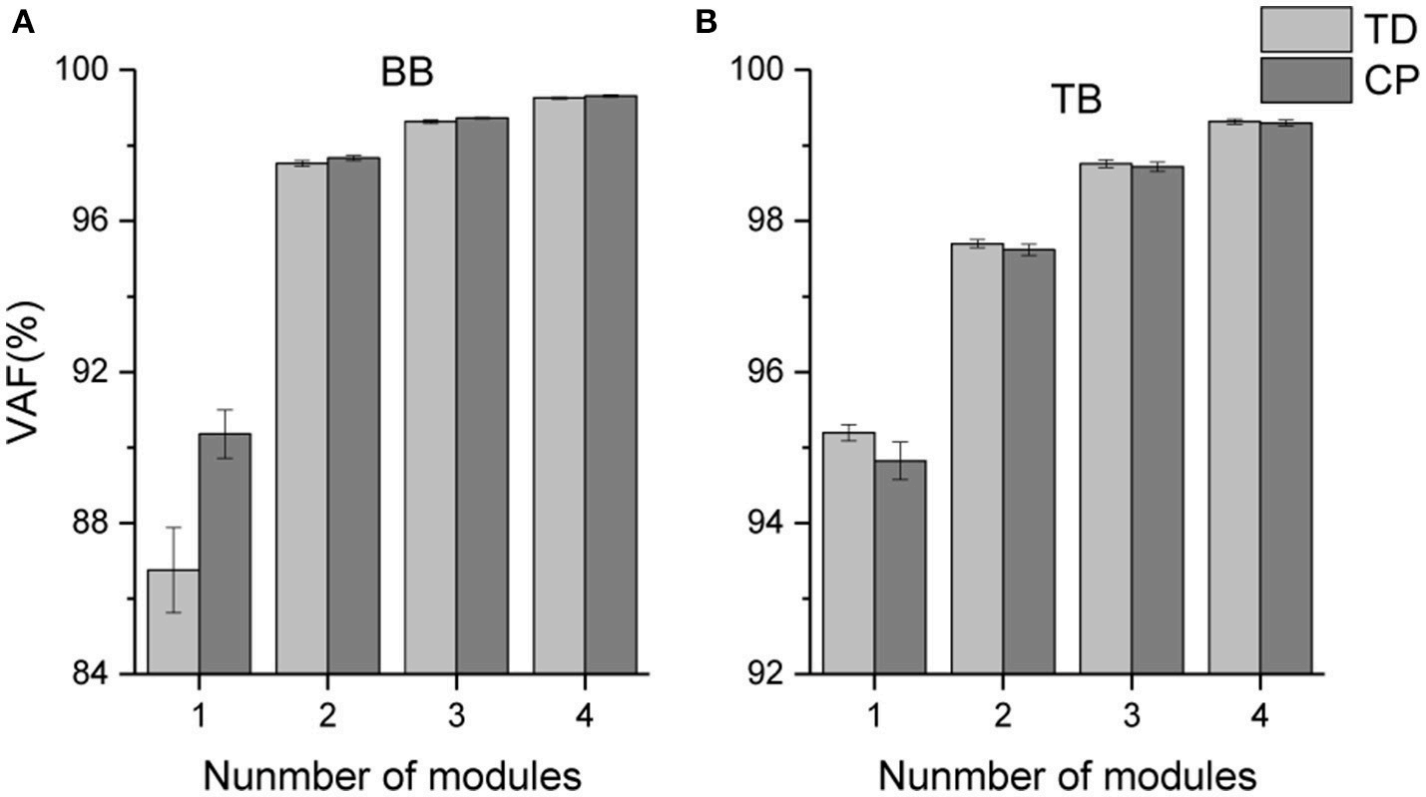
Mean VAF values corresponding to different number of oscillation synergies.

The mean VAF values acquired in the NMF decomposition of the sEMG oscillations are shown in Figure 5. For bilateral BB and TB muscles, the number of oscillation synergies (*s*) was chosen as 3 according to the selection criteria above.

**Figure 6:**
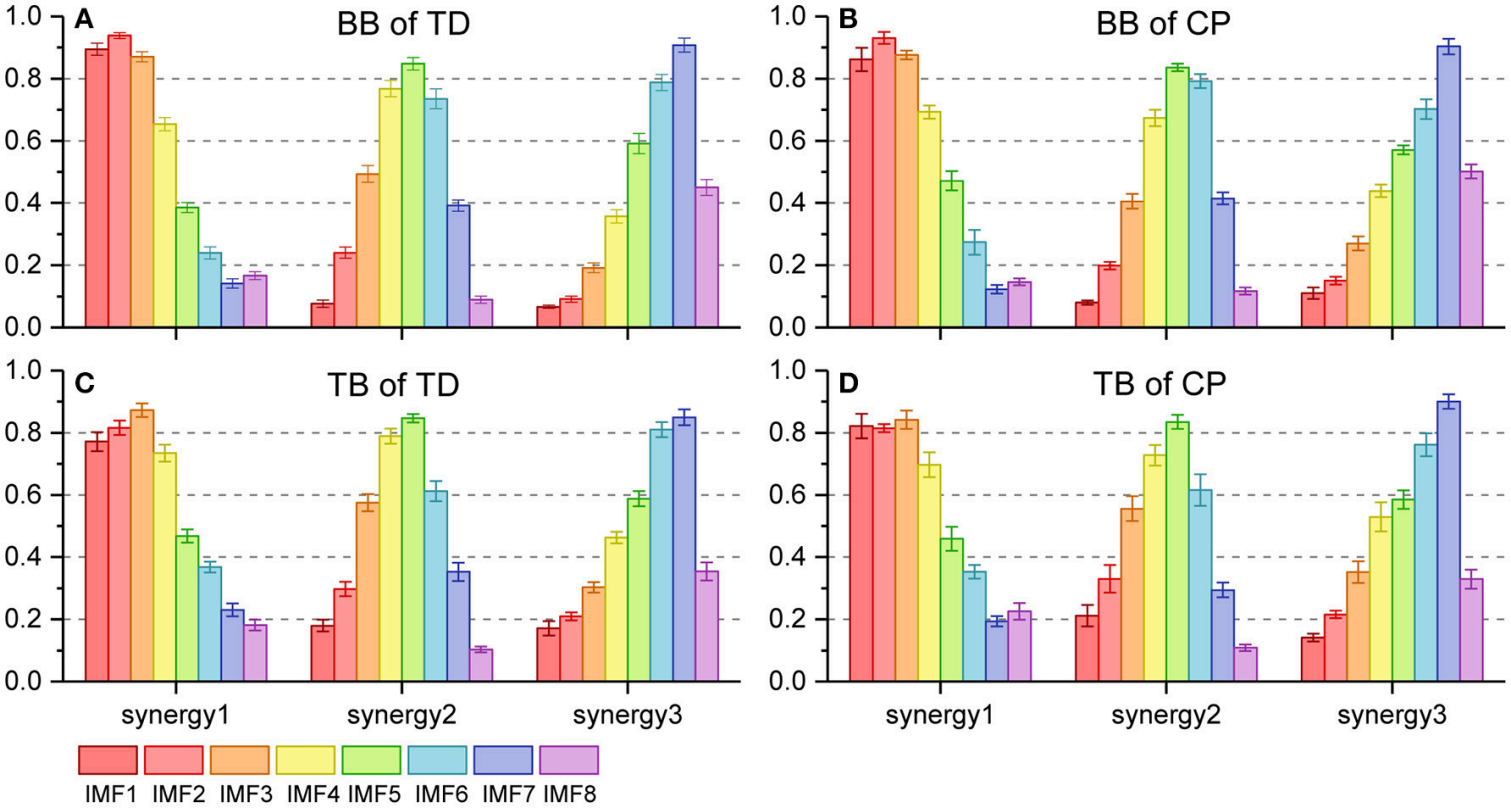
Oscillation synergies extracted from 8 sEMG IMFs of BB and TB during one crawling cycle in two groups. **(A)** BB of TD group; **(B)** BB of CP group; **(C)** TB of TD group; **(D)** TB of CP group.

Figure 6 shows the three oscillation synergies composed of the IMFs with different weights for BB and TB in TD and CP group. Each synergy of sEMG oscillation owned a dominant frequency range. The main contributors of synergy1 were IMF1, IMF2, IMF3, and IMF4, and their frequency ranged from 81 to 500 Hz (see Table 1); synergy2 was dominated with IMF4, IMF5 and IMF6, which located the frequencies from 34–189 Hz. Synergy3 was comprised of oscillations with much lower frequency, IMF6 and IMF7, and the corresponding frequency range was 23–69 Hz. Furthermore, as listed in Table 2, the Pearson's correlation coefficient of synergy 1 was more than 0.883, which indicated that the composition of IMF1~IMF8 exhibited high structural similarity for any paired muscles. The composition of IMF1~IMF8 in synergy 2 or synergy 3 showed high similarity for the BB and TB of CP and/or TD group as well, and corresponding correlation coefficients were more than 0.816 and 0.861, respectively. Altogether, there were three stable oscillation synergies of sEMG for BB and TB both in CP infants or TD infants crawling, and each synergy can be characterized with sEMG oscillation composition (i.e., IMF) of specific frequencies.

**Table 2:** Correlation coefficients between oscillation synergies.

		**Synergy1**				**Synergy2**				**Synergy3**			
		**TD**		**CP**		**TD**		**CP**		**TD**		**CP**	
		**BB**	**TB**	**BB**	**TB**	**BB**	**TB**	**BB**	**TB**	**BB**	**TB**	**BB**	**TB**
**TD**	**BB**	0.968 ± 0.003	0.912 ± 0.015	0.945 ± 0.003	0.921 ± 0.015	0.929 ± 0.006	0.894 ± 0.015	0.932 ± 0.010	0.848 ± 0.038	0.927 ± 0.006	0.892 ± 0.011	0.929 ± 0.010	0.861 ± 0.021
	**TB**		0.896 ± 0.013	0.907 ± 0.014	0.890 ± 0.012		0.897 ± 0.012	0.860 ± 0.022	0.863 ± 0.011		0.893 ± 0.010	0.883 ± 0.061	0.877 ± 0.011
**CP**	**BB**			0.931 ± 0.017	0.910 ± 0.013			0.961 ± 0.004	0.816 ± 0.053			0.938 ± 0.005	0.868 ± 0.018
	**TB**				0.883 ± 0.014				0.822 ± 0.021				0.869 ± 0.011

*Data were expressed as mean ± standard error*.

### Dynamic recruitment of oscillation synergies for crawling movement

**Figure 7:**
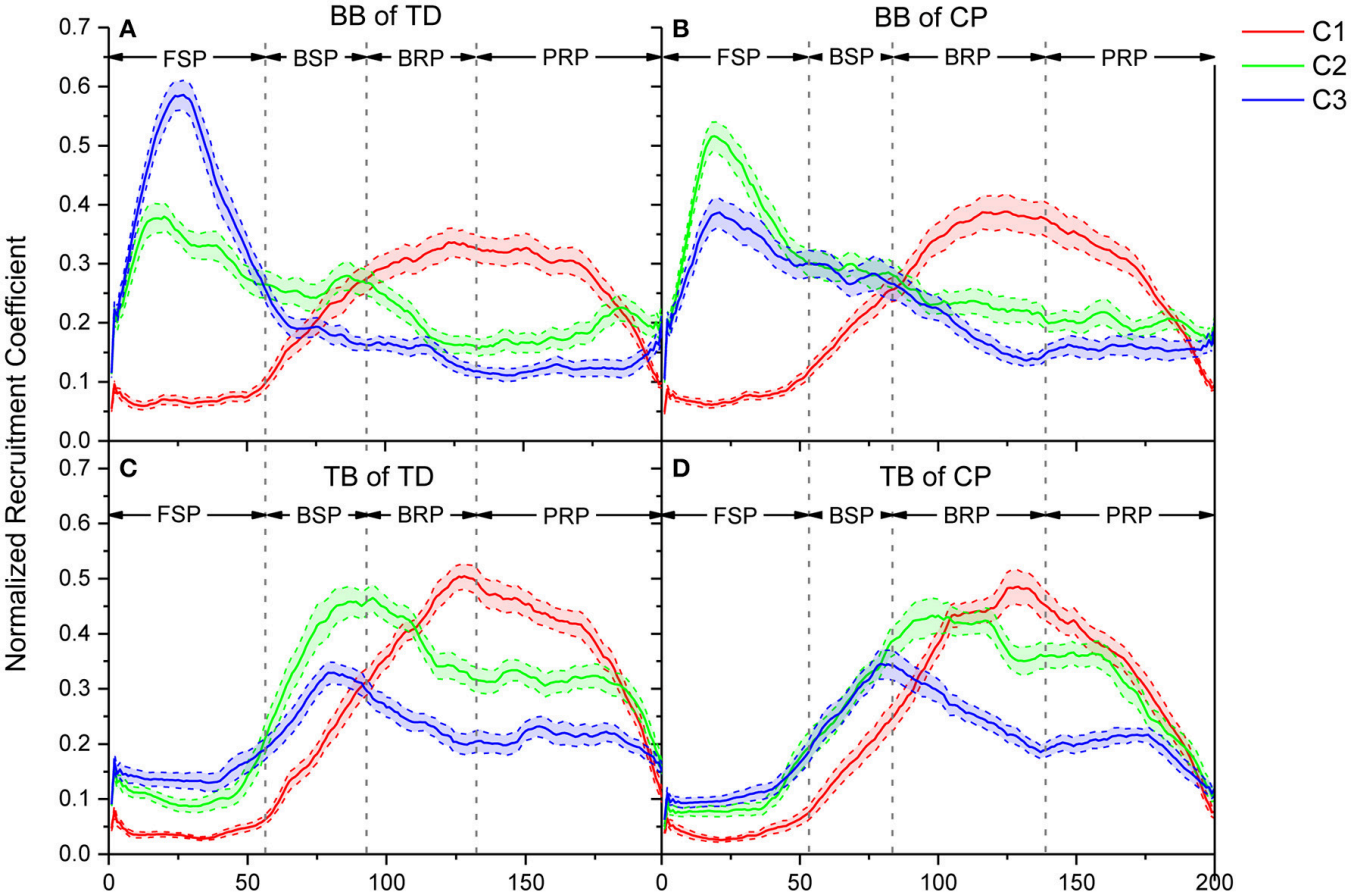
Comparison of recruitment coefficient curves for three sEMG oscillation synergies and their performance in every refined phase for TD and CP group during one crawling cycle. **(A)** BB of TD group; **(B)** BB of CP group; **(C)** TB of TD group; **(D)** TB of CP group. Red (C1), green (C2), and blue (C3) lines represent the recruitment coefficient curves of synergy1, synergy 2, and synergy 3, respectively.

The recruitment coefficient curves of sEMG oscillation synergies are illustrated in Figure 7. During a crawling cycle, each of those three oscillation synergies was dynamically recruited with time-varied coefficient curves. It can be observed, either for TD or CP infants, that they recruited the synergy 1 in BB with a low level firstly, then the recruitment reached a peak level in the second half crawling phase. Whereas the recruitment strength of synergy 2 and synergy 3 reached a peak quickly at the beginning of crawling cycle (i.e., FSP) and decreased then. For the TB, both TD and CP infants almost synchronously recruited those three synergies, and their activation level reached a peak in the middle phase of crawling (i.e., BSP and BRP). However, the motor dysfunction of CP affected the recruitment of oscillation synergies for crawling movement both in BB and TB.

### The impact of CP on the recruitment pattern of oscillation synergies

**Figure 8:**
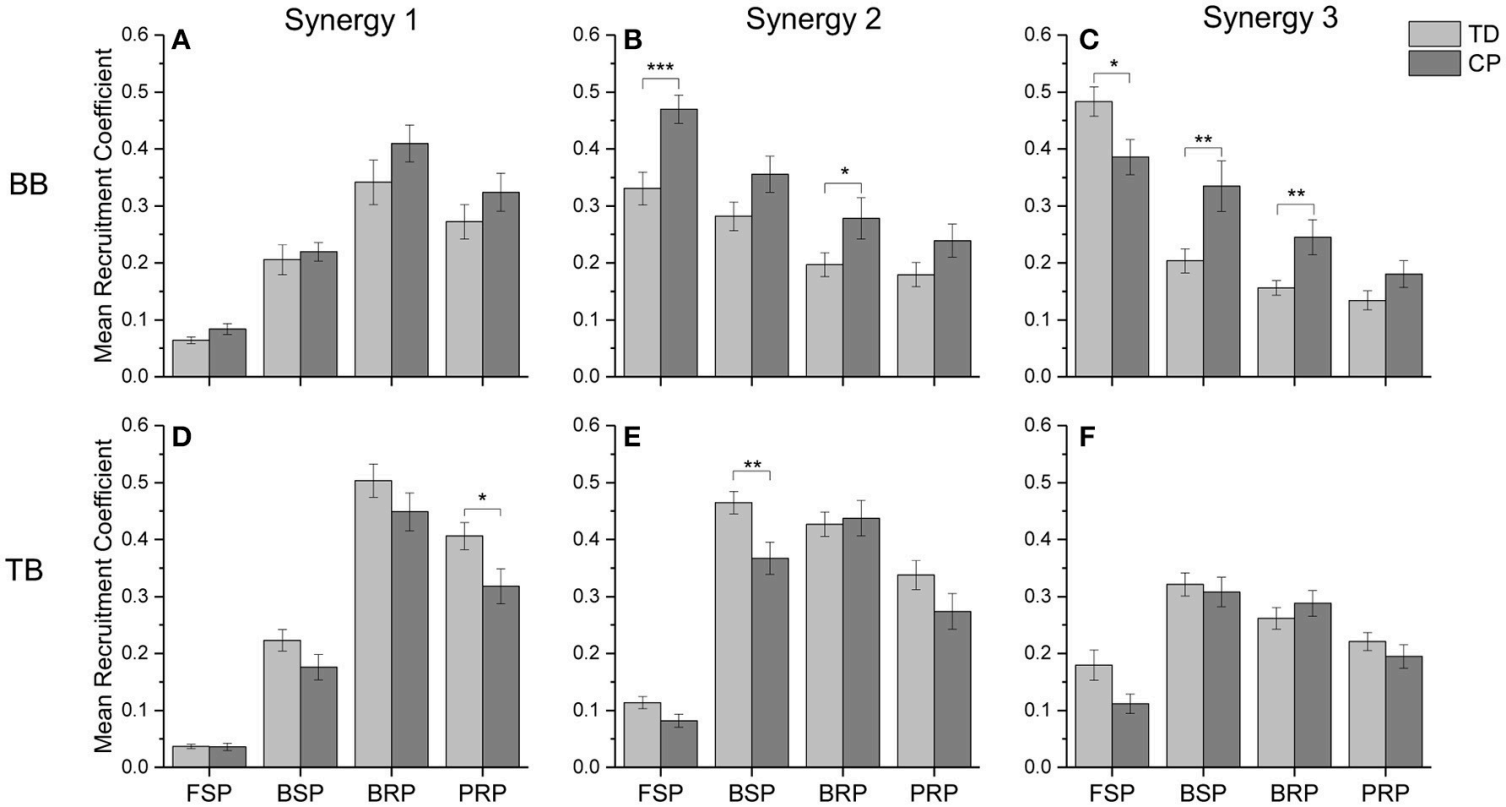
The mean recruitment coefficient of three oscillation synergies from BB and TB in two groups. **(A)** synergy 1 of BB; **(B)** synergy 2 of BB; **(C)** synergy 3 of BB; **(D)** synergy 1 of TB; **(E)** synergy 2 of TB; **(F)** synergy 3 of TB. *0.01 < *p* < 0.05, **0.005 < *p* < 0.01, ***p < 0.005.

To quantitatively evaluate the impact of CP on the recruitment pattern of oscillation synergies, the amplitude of recruitment coefficient curves was compared in the refined crawling phases of FSP, BSP, BRP, and PRP. As shown in Figure 8, CP recruited significant less synergy3 or low-frequency sEMG oscillations in BB for slow contraction in FSP crawling (Figure 8C, *p* = 0.023), and less synergy 1 or high-frequency sEMG oscillations in TB for the fast contraction in PRP crawling (Figure 8D, *p* = 0.007). During the BSP (*p* = 0.007) and BRP crawling (*p* = 0.006), CP maintained a high level of synergy3 in BB, which revealed that the sEMG oscillations with low-frequencies cannot be de-recruited effectively (Figure 8C). Furthermore, during CP crawling in FSP (*p* = 0.002) and BRP (*p* = 0.047), there was a significantly higher activation level for synergy 2 in BB (Figure 8B), whereas less synergy 2 was recruited in TB for BSP crawling (Figure 8E, *p* = 0.029).

## Discussion

To deeply explore the effect of neuromuscular damage of CP on the motor regulation, this study aimed to extract and evaluate the oscillation synergy patterns from sEMG signals with MEMD and NMF during infant crawling. The present work showed that sEMG signals contained stable structures of oscillation synergy, and the motor dysfunction of CP affected the recruitment of oscillation synergies for crawling movement both in BB and TB.

### Synergistic recruitment of sEMG oscillations for crawling movement

Crawling requires multiple skeletal muscles to participate in the regulation of limb joint flexion and extension. Muscles play different roles and their contraction patterns also vary with crawling phases. The results showed that the activation pattern of muscles can be expressed as synergistic recruitment of multi-scale sEMG oscillations. As shown in Figure 6, the activation of BB or TB muscles during crawling can be represented by the synergistic combination of the oscillation subsets with different frequency ranges, i.e., high-frequency range (synergy1), medium-frequency range (synergy2), and low-frequency range (synergy3). Moreover, as shown in Figure 7, these synergistic sEMG oscillations have been dynamically organized for infant crawling movement. Both for BB and TB, the activation level of high-frequency synergy (C1) keeps to a way of progressively developing pattern, whereas the activation level of medium- and low-frequency synergy (C2 and C3) reach a peak and then descend gradually. These results follow the principle that the recruitment of MUs has been typically graded (41) from slow to fast during dynamic contractions (42) for joint movement, and types of recruited MUs can be manifested as sEMG frequency components (17, 18). In context, the observations in our study suggest that, during the swing and stance of infant crawling, the organization of MUs activation pattern in BB and TB can be represented as synergistic sEMG oscillations, which have been dynamically regulated for different contraction status and crawling phase. Although there is some time delay for BB and TB contraction due to their different role in crawling movement, the coefficient curve for sEMG oscillations with high-, medium-, and low-frequency are alternatively enhanced to a complementary pattern among these synergies, which reveal that different types of MUs have been coordinately recruited for limb movement in crawling.

Crawling is a periodic movement of flexion-extension, in which the upper limbs postures alternate with FSP, BSP, BRP, and PRP successively. Meanwhile, BB and TB exhibit alternating coordinate relaxation and contraction. During swing, BB gradually contracts to achieve forward swing of upper limbs in FSP; slow MUs are activated, and the recruitment strength of synergy 2 and synergy 3 with lower-frequency range reach a peak. However, TB presents some time delay in crawling movement and kept extension/relaxation in FSP, and then begin contraction to swing arm backward with fast developing of high-, medium-, and low-frequency oscillations, and medium- and low-frequency oscillations reached their peak activations. To perform backward swing, BB extends in BSP as slow MUs are de-recruited, and the activation of high-frequency oscillations exceeded the medium- and low-frequency ones. During stance, both BB and TB are activated to response loading period, and fast MUs are dominantly recruited accordingly to absorb the strike shock and quickly stabilize the kinematic behavior of the joint. As shown in Figure 7, the coefficient curve of synergy 1 (C1) maintains a high level, whereas medium- and low-frequency oscillations (C2 and C3) decrease gradually. In other words, more fast MUs for high-frequency oscillations are activated to compensate the de-recruited slow MUs in stance phase of crawling, which is a mechanism for CNS timely and moderately regulating the recruitment of different types of MUs in muscles for joint movement (42).

### Abnormal recruitment pattern of oscillation synergies under motor dysfunction of CP

Motor dysfunction of cerebral palsy originates from brain injury leading to a series of neurological changes, such as reduced input to the CNS (43) and decreased activity of descending inhibitory system (44). Subsequently, motor dysfunction of CP is also manifested as abnormal myoelectric activities and abnormal movement postures due to the affected cerebral nerve in motor area (2). As illustrated in Figures 6, 7, although three types of synergistic sEMG oscillations with different frequency band are presented during CP infants crawling, there are some changes in recruitment coefficient curves, which suggest that brain injury of cerebral palsy affected the MUs recruitment. Generally, CNS employs a synergistic strategy to organize the multi-element motor system within different scales to simplify motor control (45), and the combination of motor elements with certain weights constructs motor synergy. However, motor dysfunction of CP mainly alters the organization of medium- and low-frequency oscillations of sEMG, especially the intensity proportion between medium- and low-frequency oscillations. Compared with TD, CP infant recruits less low-frequency oscillation (synergy3) during FSP crawling, whereas TB activates less medium-frequency oscillation (synergy2) during BSP crawling. It was reported that the impaired CNS of CP is unable to effectively drive low-threshold MUs (46–48), our results reveal that the inadequate recruitment of low-threshold MUs induced insufficient activation of low- and medium-frequency oscillations when BB and TB of CP infants perform flexion contraction in FSP and BSP crawling, respectively.

In addition to the aforementioned insufficient activation of low- or medium-frequency sEMG oscillations, it also can be observed that motor dysfunction of CP is unable to de-recruit those undesired sEMG oscillations. As shown in Figures 7, 8, for the BB of CP infants, the coefficient curves of synergy3 (C3) and synergy2 (C2) hold on an abnormal level in BRP crawling, and coefficient curve of synergy3 (C3) also keep on an abnormal level in BSP crawling. These results suggest that, during BRP and BSP, CP infants are unable to inhibit their low-frequency and/or medium-frequency sEMG oscillations in the BB muscles. It is accepted that different MU owns different intrinsic contraction properties, and slow MUs are activated for slow contraction and low-intensity activities, while fast MUs are recruited for fast contraction and high-intensity activities (49, 50). Central nervous system selectively recruits desired MUs and de-recruit undesired MUs to produce synergistic contraction pattern, and organizes different types of MUs at a certain activation level can improve the synergistic control for muscular activities and joint movement. For CP infant, motor dysfunction of injured motor nerve or declined activity of descending inhibitory system (31) cause an insufficient organization of different types of MUs; another observation showed that spastic diplegic CP loss the ability to fully recruit and optimally activate available motor units because of their central activation failure (39). So, our work reveal that, the synergistic recruitment of MUs can be characterized with the coordinated activation of different types of MU with appropriate proportion, and CP cannot effectively regulate the activation intensity among different MUs.

### Synergistic organization of muscle functional units exhibited by oscillation synergy patterns

The regulation of motor function by the CNS is a synergistic organization of multiple function units, which has been widely confirmed in muscle groups. Variety of methods have been used to evaluate the intensity, spectrum or frequency component of sEMG activity. However, it is still difficult to analyze the organization patterns of different types of MUs. Multi-scale oscillation modes in sEMG signals are a combination of MU activation and organization patterns. The results of this study demonstrated that the synergistic organization of different oscillations in sEMG signals can be used to evaluate the organization of MUs. In fact, sEMG frequency components are related to physiological state and have been applied to assess motor function under different conditions (23, 13, 14). However, these studies only provided an overall effect of frequency components in a certain period of time. We decomposed sEMG signals of individual muscles into multi-scale oscillations, which is correlated to MU recruitment pattern, and then, utilized NMF to extract oscillation synergies and their recruitment coefficient curves. The time-varying curves represent how CNS regulate oscillation synergies over time, which provided a novel insight to better understand the dynamic process of the CNS organizing different types of MUs.

The results of this study showed that three stable multi-scale oscillation synergies might be underlying intrinsic structure of EMG activity during crawling. The coefficient curves can further reveal the abnormal MU activation patterns during crawling in CP. Some researchers have found that motor dysfunction of CP manifested as abnormal entropy of multi-scale oscillations of sEMG activities in CP, and a major peripheral cause of these abnormalities is abnormal MU recruitment (10, 16). This study revealed that motor dysfunction of CP also manifested as abnormal recruitment of oscillation synergies. For CP, the motor dysfunction is caused by original brain injury and resulted abnormal descending motor commands. It can be observed that, in CP group, the oscillation synergies with low-frequency range (synergy3) were insufficiently recruited when BB muscles performed slow contraction during swing, on other hand, they insufficiently de-recruited the oscillation synergies with low-frequency range (synergy2 and 3) when BB extended and relaxed in BSP and BRP. These results suggested the recruitment coefficient curves of oscillation synergies can exhibit the time-varying organization of sEMG oscillations modulated by CNS, and the curves also can reveal abnormal motor control caused by neuromuscular deficits.

## Conclusion

This is the first time to adopt the synergistic mechanism of motor function to characterize the recruitment pattern of sEMG oscillations. The present results reveal that, the myoelectric activities of a muscle can be manifested as sEMG oscillation synergies with different frequency ranges, and motor dysfunction of CP degrade the synergistic recruitment of sEMG oscillations due to the impaired CNS regulation and destroyed MU/muscle fiber. Our preliminary work suggests that, the synergistic organization for motor control can be manifested rather than muscle group, there are oscillation synergies in sEMG signals. Also, the time-varying coefficient curve of sEMG oscillation synergies is a potential index to evaluate the abnormal recruitment of different types of MUs affected by CP disorders. In future work, we would further investigate the sEMG oscillation synergies in other muscles of upper and lower limbs, and the body size of test sample should be enlarged to verify the influence of CP on the synergistic recruitment of sEMG oscillations.

## Ethics statement

This study is under the approval of children's hospital of Chongqing medical university, and all the tests are conducted in the department of rehabilitation center, children's hospital of Chongqing medical university.

## Author contributions

ZG analyzed the data and signals. ZG, LC, and WH drafted and revised the work. XW, NX, WJ, and WH interpreted the data. ZG, QX, and YL collected the data. XW and WH proposed the research topic. WH organized the data collection, signal processing, and manuscript draft.

### Conflict of interest statement

The authors declare that the research was conducted in the absence of any commercial or financial relationships that could be construed as a potential conflict of interest.
